# Cognitive and motor behaviors in Alzheimer's, Parkinson's, and Healthy individuals during the ViewMind 3‐D Task

**DOI:** 10.1002/alz70856_106668

**Published:** 2026-01-08

**Authors:** Gerardo Fernández, Luis Mendez, David Aguillon, David Orozco, Ramiro Linares, Gustavo Sgrilli, Gustavo Echeverria, Danilo Verge, Mario A A Parra

**Affiliations:** ^1^ ViewMind Inc., New York, NY, USA; ^2^ Grupo de Neurociencias de Antioquia, Universidad de Antioquia, Medellin, Colombia; ^3^ Clínica Privada Bahiense, Bahía Blanca, Argentina; ^4^ Axis Neurociencias, Bahia Blanca, Argentina; ^5^ AXIS, Bahia Blanca, San Martín 724, Argentina; ^6^ ViewMind, NY, NY, USA; ^7^ University of Strathcylde, Glasgow, Glasgow, United Kingdom

## Abstract

**Background:**

Oculomotor behaviors linked to cognitive performance can differentiate between the trajectories of neurodegeneration (ND) that lead to Alzheimer's disease (AD) (Fernández (Fernandez et al., 2022) or Parkinson's Disease (PD) (Mosimann et al., 2005). We investigated whether ViewMind technology, which combines Virtual Reality, Hand Movement Sensors (HMS), eye tracking (ET) and 3D Spheres Touching Task (3DT) identifies different cognitive and motor behaviors among symptomatic (AD) and asymptomatic carriers (ASYMPTAD) of the mutation E280A‐PSEN1 from Antioquia, Colombia, PD patients (without and with MCI – PDMCI), and Controls (HC).

**Methods:**

We recruited 76 ASYMPTAD (age 32.5±8.1), 16 AD (age 66.6±8.5), 45 PD (age 64.5±9.4, 10 PDMCI (age 68.2±9.8) and 62 HC (age 34±10.2). All performed standard neuropsychological tests and the 3DT. The task assesses the ability to detect 3D visual targets (i.e., visual accuracy and reaction time), to track these targets as they move from the background to the subject (i.e., tracking accuracy), and hand movement initiation towards targets (i.e., hand accuracy and reaction time).

**Results:**

Tracking accuracy, hand reaction time, and visual reaction time as a function of the number of touched targets were more affected in AD, PD, and PDMCI patients when compared to ASYMPTAD and HC. ASYMPTAD and HC reached the most significant number of targets. Mean tracking accuracy followed the pattern PDMCI > PD > AD > HC & ASYMPTAD (*p* <0.0001). Mean hand reaction time followed the pattern PDMCI > AD > PD > HC & ASYMPTAD (*p* <0.0001). Mean visual reaction time followed the pattern AD > PDMCI > PD > HC & ASYMPTAD (*p* = 0.006).

**Conclusion:**

ViewMind's 3DT identifies cognitive and motor profiles across neurodegenerative diseases. Impairments were apparent in and discriminable between clinical groups with (PD) or without (AD) predominant movement disorders and lacking in non‐clinical samples (HC & ASYMPTAD). Available assessments cannot identify the interplay of motor and cognitive control during ongoing ecologically valid tasks. This highlights the role of this novel technology in refining theories and applications of visual‐motor integration tasks in ND diseases.

**References**

1. Fernandez et al. (2022). Alzheimer's & Dementia, 18(S5), e066784.

2. Mosimann et al. (2005). Brain, 128: 1267–76.

